# Image segmentation using transfer learning and Fast R-CNN for diabetic foot wound treatments

**DOI:** 10.3389/fpubh.2022.969846

**Published:** 2022-09-20

**Authors:** Huang-Nan Huang, Tianyi Zhang, Chao-Tung Yang, Yi-Jing Sheen, Hsian-Min Chen, Chur-Jen Chen, Meng-Wen Tseng

**Affiliations:** ^1^Department of Applied Mathematics, Tunghai University, Taichung, Taiwan; ^2^Department of Computer Science, Tunghai University, Taichung, Taiwan; ^3^Research Center for Smart Sustainable Circular Economy, Tunghai University, Taichung, Taiwan; ^4^Division of Endocrinology and Metabolism, Department of Internal Medicine, Taichung Veterans General Hospital, Taichung, Taiwan; ^5^Department of Medical Research, Center for Quantitative Imaging in Medicine (CQUIM), Taichung Veterans General Hospital, Taichung, Taiwan

**Keywords:** diabetic foot, deep neural network, wound treatment, Fast R-CNN, transfer learning, GrabCut, SURF

## Abstract

Diabetic foot ulcers (DFUs) are considered the most challenging forms of chronic ulcerations to handle their multifactorial nature. It is necessary to establish a comprehensive treatment plan, accurate, and systematic evaluation of a patient with a DFU. This paper proposed an image recognition of diabetic foot wounds to support the effective execution of the treatment plan. In the severity of a diabetic foot ulcer, we refer to the current qualitative evaluation method commonly used in clinical practice, developed by the International Working Group on the Diabetic Foot: PEDIS index, and the evaluation made by physicians. The deep neural network, convolutional neural network, object recognition, and other technologies are applied to analyze the classification, location, and size of wounds by image analysis technology. The image features are labeled with the help of the physician. The Object Detection Fast R-CNN method is applied to these wound images to build and train machine learning modules and evaluate their effectiveness. In the assessment accuracy, it can be indicated that the wound image detection data can be as high as 90%.

## 1. Introduction

According to 2021 data from the International Diabetes Federation (IDF), the global diabetics' prevalence in 20–79 years old in 2021 to be 10.5% (536.6 million people), rising rapidly to 12.2% (783.2 million) in 2,045 (1). Chronic diseases such as diabetes are complex and challenging to control. If poorly controlled, many serious complications will result in the paper (2), such as diabetic foot. The foot wound is difficult to heal in the initial stage, and if not correctly handled, ulcerated amputation will result. According to previous studies, about 25~34% of diabetic patients have at least one-foot ulcer in their lifetime, and the amputation rate is 15 times higher than that of non-diabetic patients (3). For each additional year of diabetes history (4), the amputation risk was 1.06 to 1.15 times greater. Regarding economic status and environmental factors, the study of Wrobel et al. (5) pointed out that lower amputation in the United States is significantly different in different regions, which may be related to the different allocation of medical resources in different regions and the different socio-economic statuses of patients.

In 2001, the Bureau of National Health Insurance (BNHI) promoted diabetes Pay for Performance (P4P) payment method, aiming at four indicators of physician care for patients: Complete follow-up rate of patients (more than three follow-up visits per year), reasonable control rate of HbA1C (HbA1C < 7%), poor control rate of HbA1C (HbA1C > 9.5%) and LDL bad control rate (HbA1C > 9.5%) were evaluated. The purpose was to induce medical providers to improve the quality of medical care for diabetes by changing the payment system to control the disease. Studies have shown that the implementation of DIABETES P4P can improve the medical treatment procedures and clinical care quality, and the outcomes of diabetes patients with P4P are better than those without P4P in terms of compliance with care guidance, self-care ability, physiological indicators, and the implementation rate of clinical monitoring indicators, indicating the effectiveness of diabetes P4P in Taiwan. The incidence of the diabetic foot has fallen, but about 3,000 diabetics still progress to needing an amputation each year (6).

A large proportion of medical records are presented in written form. However, studies using artificial intelligence to process text to supplement medical advice are still subject to considerable change and difficulty due to reasons such as physician autonomy (7, 8). Along with the rapidly increasing number of people with diabetes, we will try to develop an auxiliary diagnostic system by collecting diabetic foot wound pictures and developing the future of image SOP procedures. Then, using machine learning tools to achieve the automated assessment of the wound change and artificial intelligence progress to deal with the medical record. The machine learning models will process information application in the medical field in real-time auxiliary diagnosis, and doctors determine possible diseases to improve the efficiency of medical diagnosis (9–11).

According to Oliver and Mutluoglu (12), patients with poorly treated diabetes mellitus may get diabetic foot ulcers as one of their most common consequences. Poor foot treatment, underlying neuropathy, poor glycemic control, or peripheral vascular disease are the usual causes. Amputations of the lower extremities and foot osteomyelitis are also frequently caused by it. These ulcers typically develop in the parts of the foot that experience repeated trauma and pressure feelings. The most typical infectious organism is Staphylococcus. A multidisciplinary approach will yield the best results because the condition is frequently persistent. The United States study results, diabetic foot ulcers or amputations will cause severe personal, family, and social burdens. They found that the amputation of diabetes patients, medical expenses per week than peripheral neuropathy patients health spending a week, ten thousand times higher than that of Britain in 2010–2011. Diabetes health spending is as high as 6.4 to 660 million pounds. Therefore, effectively evaluating and classifying diabetic foot wounds for the early stage is crucial to mastering the treatment plan and effectively using medical resources. In addition, treating diabetic foot requires the intervention of a multi-disciplinary team, and the treatment will take a long time. The collation and recording of relevant images will also help the communication between various departments and the evaluation of wound recovery.

The system aims to analyze the degree of change of the wound (such as wound length, width, area, perimeter, geometry, and other related information). In this case, the process started with collecting photos of diabetic foot wounds and related image processing techniques and cooperating with clinicians. The essential parts are to discuss the formulation of SOP procedures for future wound image shooting, fix the shooting orientation, set lens distance, and set the reference rule for future large-scale data analysis and research. The system will have two aspects: classify the disease based on the CNN-based transfer learning from the foot image of the DFU patient (13). Another is to extract and write the characteristics of the wound area, shape, and circumference of the DFU patient into the electronic case system as a critical parameter for the evaluation of debridement treatment. Then, the back-end auxiliary physician diagnosis system uses these recognition results. The objectives of this study are listed as follows.

1. Pre-process the data, including classification, data augmentation, image annotation, and cutting data set to understand the data's correlation.
2. Use transfer learning to build a machine learning module and adjust the parameters to construct a model suitable for face symmetry data and train it.
3. Evaluate training results, compare method training accuracy under different parameters, and determine suitable training parameters.

Transfer learning uses the existing convolutional neural network that has been trained in the past to migrate to the target field so that the target field can have a better learning effect (14–16). Therefore, a model for recognizing the face's symmetry has been successfully trained through parameter adjustment and photo selection, but the accuracy can also be improved through some approaches.

## 2. Background review and related work

### 2.1. Object detection

The method of Fast R-CNN in Object Detection performs object detection on diabetic foot wounds, in addition to identifying all the objects that appear from the image and marking the position of the object (17–19). Fast R-CNN uses the concept of Sliding Windows, uses a fixed-size classifier to scan the entire photo one by one, and delivers the extracted image to the convolutional neural network for identification and classification, and through the non-stop change of the size of the classifier to recognize objects of different sizes.

- It produces about 2,000 image regions (Region Proposals) where objects may appear.
- Through a pre-trained convolutional neural network model, ResNet is used to extract features.
- Use the classifier to distinguish whether it is an object or a background.
- Finally, the position of the bounding box is corrected *via* the linear regression model.

### 2.2. Transfer learning

Recently, in medical imaging, due to the continuous accumulation of large amounts of medical data, developing these data to alleviate medical-related problems has become a topic of concern. Therefore, deep learning networks are used in medical imaging. Applications are gradually emerging. Medical imaging company, Enlitic conducts deep learning training through many medical images and diagnostic data to achieve a fast and accurate medical diagnosis. However, researchers have found that with the development of research, problems such as insufficient images, missing labels, and distribution drift have gradually become obstacles to applying deep learning to medical imaging. Therefore, this paper uses deep learning-based transfer learning to compensate for deep learning.

Transfer learning is a method used for machine learning. It is to transfer the knowledge of the original field to a new field to achieve good learning results. We can divide the data for transfer learning into two categories: source data and the other is target data. Source data refers to additional data and is not directly related to the task to be solved, while target data is directly related to the task. According to whether the two samples have the same purpose, transfer learning can be divided into inductive transfer learning, direct push transfer learning, and unsupervised transfer learning. According to the technology used in the transfer learning method, the transfer learning method can be roughly divided. The top is divided into:

1. Transfer learning based on feature selection.
2. Transfer learning based on feature mapping.
3. Weight-based transfer learning.

### 2.3. Related works

Zahia et al. (20) proposed a handwritten Hindi numeral digit recognition structure based on the Convolutional Neural Network (CNN) with the RMSprop optimization technique. The network's structural architecture consists of a convolution (Conv2D) sheet, a sheet of pooling (MaxPool2D), a flattening layer, and two fully linked layers. A sliding window functionality is added to a numerical image matrix. It tested the scheme on 20,000 handwritten samples of Hindi numerals from the Kaggle dataset. They achieved a 99.85% recognition rate by the proposed RMSprop (Root Mean Square Propagation) Optimization Convolutional Neural Network system, which is very promising.

Blanco et al. (21) demonstrated a completely automated fine-tuning of CNNs for image retrieval on a wide array of unordered images. The collection of training data is driven by reconstructed 3D models obtained through state-of-the-art retrieval and structure-from-motion methods. They proposed a new Generalized-Mean (GeM) trainable pooling layer that generalizes max and average pooling and demonstrates that it improves recovery efficiency. The VGG network's implementation of the proposed approach achieves state-of-the-art efficiency on the standard benchmarks: Oxford Houses, Paris, and Holidays datasets.

Bria et al. (22) applied the GPDS Synthetic Signature Database to identify 1,000 users' signatures, each with 24 original (or genuine) signatures and 30 forged (or fake) signatures. In addition, two common CNN variants of the GoogLeNet architecture, namely, Inception-v1 and Inception-v3, were used. Inception-v1 obtained an EER of as little as 17 for 20 users, while EER for Inception-v3 obtained 24 for 20 users, which is a decent measure compared to previous literature works. In comparison, Inception-v3 performed better in the ImageNet image classification challenge. Inception-v1 performed the classification task better than Inception-v3 in the case of 2D images of signatures. It is also recognized that Inception-v1 spent less time training, as it had a smaller number of operations than Inception-v3.

Zhang et al. (23) present that using 3D roto-translation group convolutions instead of standard translational convolutions, the sample complexity of CNNs can be significantly improved. 3D CNNs with community convolutions (3D G-CNNs) have been applied to the problem of false-positive reduction of pulmonary nodule detection in CT scans and be significantly more effective in terms of precision, sensitivity to malignant nodules, and speed of convergence compared to a comparable and robust baseline architecture with regular convolutions, comprehensive data augmentation, and similar nu-convolutions. The G-CNN reached an FROC score similar to the CNN trained on 10 times more data for each data set size evaluated.

Pakhomov et al. (24) indicate that using machine learning to identify foot examination findings from clinical reports is a viable alternative to manual review and warrants further investigation. Wang et al. (25) used SVM based classification with local scale-invariant feature transform (SIFT) features to detect diabetic foot ulcers. In Goyal et al. (26) demonstratesd the potential of fully convolutional networks (FCN) in DFU segmentation, which can be further improved with a larger dataset.

Cassidy et al. (27) describe the DFUC 2020 Dataset and apply various types of CNN to analyze DFU photographs with references therein reviewing related works. This dataset is adopted by “Diabetic Foot Ulcers Grand Challenge 2020” Challenge, accepted for International Conference on Medical Image Computing and Computer Assisted Intervention (28). Later on, Cassidy et al. (29) propose a mobile and cloud-based framework for the automatic detection of diabetic foot ulcers with a deep CNN deployed to photographs of patients feet for inference to detect the presence of diabetic foot ulcers. Unfortunately, the DFU photographs in DFUC 2020 Dataset contain the wound with compatible size but in real clinic applications, the size of wounds are very different.

## 3. System design and implementation

This system provides a record and analysis platform for medical staff. The doctor uploads the patient's wound photo to the system. After analysis, the system will display the patient's wound's specific category and provide data such as the wound size and circumference. Therefore, the doctor can judge the wound's condition and apply machine learning. For the diagnosis and treatment of diabetic foot ulcers, at the same time, if the patient undergoes multiple diagnoses and treatment, it can be passed. The circumference and area of several judgments of the wound calculate whether the wound has healed or deteriorated. Our system assists physicians in making preliminary medical assessments of the degree of ulcers and provides objective data references (30).

### 3.1. System architecture

**Figure 1:**
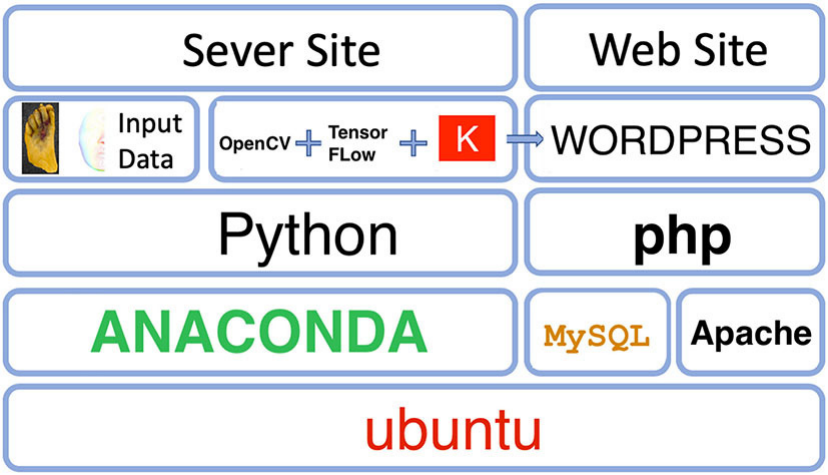
System architecture diagram.

**Figure 2:**
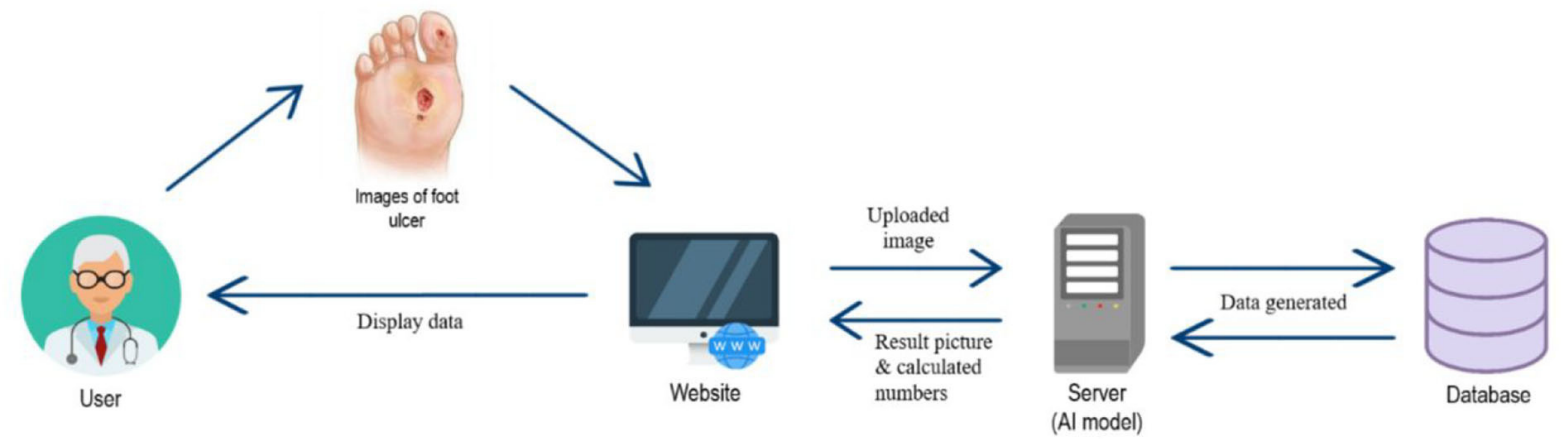
Data flow diagram.

The system in this article is built on the Ubuntu 18.04 operating system. The diabetic foot wound data and face symmetry data images will be pre-processed through OpenCV and Keras, and Tensorflow will be used to train to generate machine learning modules. The generated data will be stored in the database through MySQL and presented on the web page through PHP. Figure 1 is a system architecture diagram. Figure 2 shows a data flow diagram.

### 3.2. Hardware and software specifications

The following list shows the hardware, software, and technology used in this paper.

**Hardware**

- CPU Info: Intel (R) Core (TM)
- i7-3970X CPU @ 3.50GHz
- Memory (RAM): 64GB
- Hard Disks: 1TB
- Display Adapters: NVIDIA® GeForce®
- RTX 2080 Ti
- CUDA: Toolkit 10.0
- cuDNN: cuDNN Release v7.4.1

**Software**

- Ubuntu v18.04.1
- MySQL v5.7.27
- Python v3.6.8
- PHP v7.2.24
- Jupyter Notebook v4.4.0
- Sublime Text 3

**Technology**

- TensorFlow-GPU v1.14.0
- Keras v2.2.4
- OpenCV v4.1.0
- HTML5
- CSS3
- JavaScript
- WordPress v5.2.4
- Bootstrap v4.3.1
- jQuery v1.12.4

### 3.3. Fast R-CNN model

We selected three Fast RCNN modules for migration, namely Inception V2-coco, Kitti-Trained models (ResNet101), and iNaturalist Species-trained models (ResNet101). The most accurate deep learning module to identify diabetic foot wounds by comparing mAP accuracy, recognition speed, and parameter adjustment.

1. iNaturalist Species-trained models and Kitti-Trained models. Both of them use the ResNet deep neural network to search the image's features. ResNet can make the structure reach 1,000 layers through residual function, and at the same time make the classification effect of the structure reach an excellent effect. The core idea of ResNet (31) is to introduce an identity shortcut connection structure, directly skip one or more layers. For a stacked layer structure (made of several layers stacked), when the input is *x*, the learned feature is denoted as *H*(*x*). Now, we hope it can learn the residual *F*(*x*) = *H*(*x*) − *x*, so that the original learned feature is then given by *F*(*x*) + *x*. It is because the residual learning function is more accessible to estimate than the direct feature learning function. When the residual is 0, the residual stacking layer does identity mapping at this time, and at least the network performance will not decrease. The residual will not be 0, enabling the stacking layer to learn new features based on the input features.

Let *X*_0_ denote the original image. So as to have better performance, the residual unit can be expressed as


(1) $$\begin{array}{r} y_{l}=h\left(X_{l}\right)+F\left(X_{l},W_{l}\right), \end{array}$$

(2) $$\begin{array}{r} X_{l+1}=f\left(y_{l}\right), \end{array}$$

where *X*_*l*_ and *X*_*l*+1_, respectively, represent the input and output of the *l*-th residual unit, and *W*_*l*_ = {*W*_*l, k*_|1 ≤ *k* ≤ *K*} is a set of weights (and biases) associated with the *l*-th residual unit, and *K* is the number of layers in a residual unit [*K* is 2 or 3 in He et al. (31)]. Here *F* is a residual function. As discussed in He et al. (31), the function *h* is an identity mapping: *h*(*X*_*l*_) = *X*_*l*_, and *f* is a ReLU function.

If *f* is assumed also an identity mapping, i.e., *X*_*l*+1_ ≡ *y*_*l*_, then it follows that


$$X_{l+1}=X_{l}+F\left(X_{l},W_{l}\right).$$

Recursively, the learning characteristics from the shallow layer *l* to the deep layer *L* are then described by


(3) $$\begin{array}{r} X_{L}=X_{l}+\overset{L-1}{\underset{i=l}{\sum }}F\left(X_{i},W_{i}\right) \end{array}$$

This equation implies an excellent backward propagation property. Denoting the loss function as E and its gradient is computed by using Chain Rule:


(4) $$\begin{array}{r} \frac{\partial E}{\partial X_{l}}=\frac{\partial E}{\partial X_{L}}\cdot \frac{\partial X_{L}}{\partial X_{l}}=\frac{\partial E}{\partial X_{L}}\cdot \left(1+\frac{\partial }{\partial X_{l}}\overset{L-1}{\underset{i=1}{\sum }}F\left(X_{i},W_{i}\right)\right) \end{array}$$

As pointed out by He et al. (32), Equation (4) consists of two parts. The first part indicates that it propagates information directly without concerning any weight layers. The second term is inside parentheses, which indicates the short-circuit mechanism can propagate the gradient without loss, and the other residual gradient needs to pass through the weight layers. Because in general, the term $\frac{\partial }{\partial X_{l}}\overset{L-1}{\underset{i=l}{\sum }}F$ cannot be always −1 for all samples in a mini-batch. It implies that the gradient of a layer does not vanish even when the weights are arbitrarily small. So, the residual function (*F*) learning will be easier than the feature function (*H*) learning.

iNaturalist Species-trained model resnet101_fgvc: Using the photos taken by ordinary people to identify unnatural or particular species accurately. It is a module developed by developers to identify unnatural creatures and supports ordinary shooting methods, which can be used to train ordinary people to shoot pictures, and uncommon creatures of low-quality exhibit strange shapes or other characteristics. Kitti-Trained models (ResNet101) are also based on ResNet models, with very high accuracy and speed. Kitti models have an excellent advantage in identifying cars in traffic. The same applies to the identification of other objects.

2. Inception V2-coco

GoogLeNet's Inception architecture can achieve very good performance under strict memory and computational budget constraints. Inception is less computationally intensive than VGG but has higher performance. It allows Inception to be used in scenarios with big data (less inference time) or mobile environments (with limited memory and computing power). Inception's design philosophy is

1. Avoid representation bottlenecks, especially in front of the network. The size of activations should be gradually reduced.
2. Higher-dimensional representation is easier to handle and easier to train (convergence).
3. Spatial aggregation on low-dimensional embedded space hardly affects representation capability. The explanation for this is that there is a strong correlation between connected neurons, and there is a redundancy of information.
4. Balance the width and depth of the network. The balance between the two leads to better performance.

### 3.4. GoogLeNet transfer learning

GoogLeNet performance primarily comes from the widespread use of dimensionality reduction. For example, a 1 x 1 convolution follows a 3 x 3 convolution. In the visual task, we hope that the nearby activations are highly relevant, reducing dimensionality before aggregation. Solve the large filter volume integral into multiple small filter convolutions. This decomposition can dissociate the parameters. Therefore, the training speed will be faster.

1. Use a smaller convolution layer
  The inception model splits 5 × 5 into two 3 × 3's, the same before and after. The concatenation of two 3 × 3 convolutions is more powerful than the representation of a 5 x 5 convolution. Also, the decomposition after the use of an activation function increased the linear ability. The ratio of the number of parameters of two 3 × 3 convolutions and one 5 × 5 convolution is (9 + 9)/25, which reduces the number of parameters by 28%. There was also a 28% reduction in computation.
2. Efficient reduction of the size of the feature map
  In general, convolutional neural networks use some pooling operations to reduce the feature maps' grid size. The activation dimension needs to be increased before maximum, or average pooling is applied to avoid representation bottlenecks. For example, there is a dxd feature map of k channel. If we want to get a (d/2) x (d/2) feature map of a 2k channel, we first need to carry out the convolution of 2k channels with the stride of 1 and then apply another pooling. Inception's method to reduce the feature map's size: use a parallel (Conv + pooling stride of both paths is 2) to achieve. It does not require much performance and avoids representation bottlenecks.

### 3.5. Image-assisted diagnosis of diabetic foot wounds

#### 3.5.1. Applicable people

Medical staff including assist doctors and nursing staff quickly judge wounds and provide objective data when working on medical reports. The basis for making judgments on the classification of wounds will also be in the future to combine data sets with electronic medical records.

#### 3.5.2. System functional requirements

To meet this system's requirement, we will upload images, analyze the degree of wounds, export analysis data, and delete images. The related functions are briefly described in a list.

1. User settings
  After the user has logged in to the system, they can change personal basic data by changing the user social function.
2. File upload and analysis
  When the user has logged in to the system, they can enter the file upload page to upload the file. After the image is uploaded, the text description can be edited and the result can be viewed after analysis.
3. The file download
  Enters the post-analysis result page, and the page will display related information such as the number of images received and the x y position value. The user can click the file download to download the analysis result.
4. Picture library query
  After users upload images, they can enter the picture library to view uploaded pictures and information.
5. File deletion
  After clicking the file in the picture library, the delete file button will appear in the list below. Click to delete the file.
6. Maintain patient data
  Users who can create individual patient data and make changes or edits.

#### 3.5.3. Data classification and preprocessing

**Figure 3:**
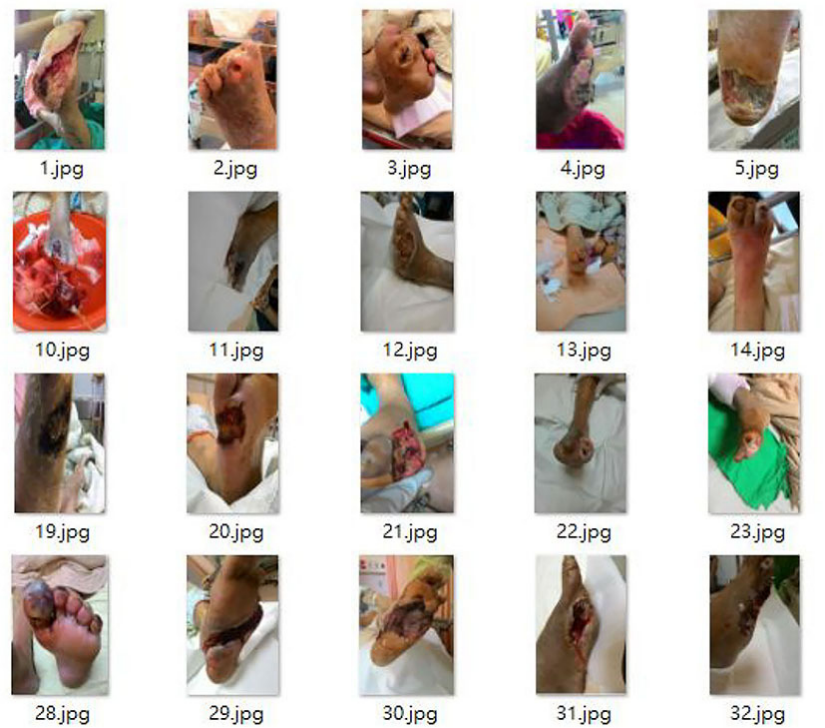
Diabetic foot wound data.

The data used in this system comes from Taichung Rongmin General Hospital and is provided by an endocrinology metabolism physician. It classifies diabetic foot wounds, including ulcer wounds (ulceration) and blood vessels blocked feet Blocked blood vessels and suture wounds. In this system, we added Rongmin General Hospital's mark to assist in subsequent judgments. Figure 3 shows the original data of diabetic foot wounds (33).

Several original files are 318 Blocked blood vessels, 64 Suture, and 345 Ulceration. After reviewing the number of wound types included in the image data, to ensure that there can be more data for the learning process's characteristics, we first pre-process the image data and augment the data set. The original insufficient image data set was processed by Keras to expand the image back and forth, upside down, displacement, and distortion, from the original total of 727 images to 900 images of each type. A total of 3,600 pictures are used as training materials. Among them, 700 images of each classification of 900 are used as Train Data, and the other 200 are used as Test Data.

#### 3.5.4. Data label and CSV file generation

**Figure 4:**
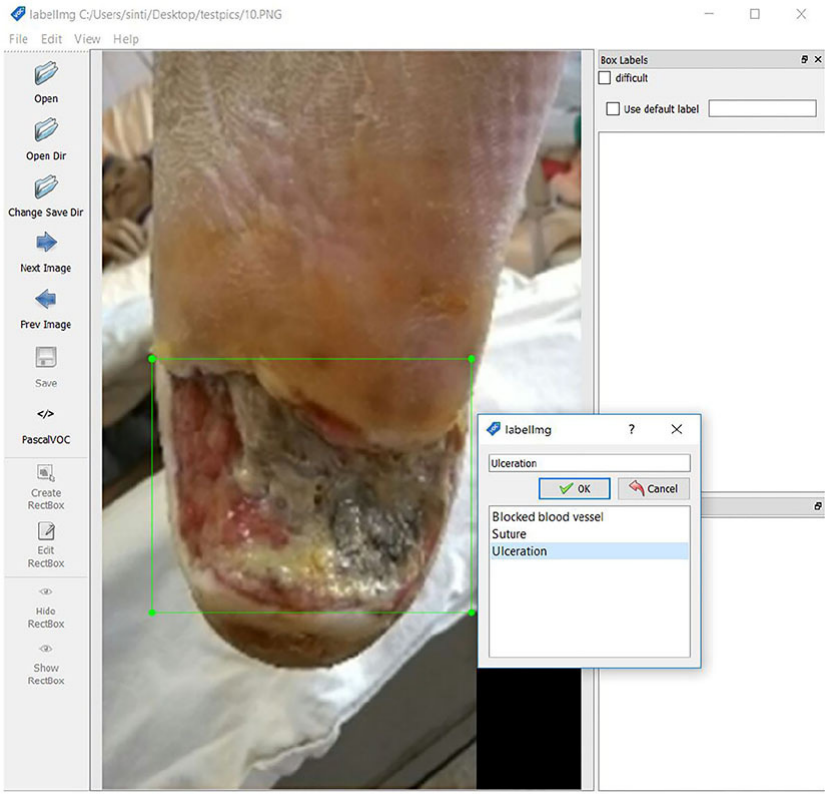
Data labeling.

We use LabelImg software to mark the amplified data set as the wound's target and specify the classification. After marking 3,600 image files, the XML data file with the target marked coordinate value is processed to generate a CSV file, and the data preparation before training is completed. Figure 4 shows the data labeling procedure.

#### 3.5.5. Module selection and adjustment of model parameters

After pre-processing the data, we start building a deep learning model. We use Tensorflow Object Detection to train diabetic foot image data and use Tensorflow as the base to test the object detection of diabetic foot wounds using the Fast R-CNN method and identify the target from the image marking the position of the object Output coordinates. R-CNN uses the concept of Sliding Windows, uses a fixed-size classifier to scan the entire photo one by one, and delivers the extracted images to the convolutional neural network for recognition and classification and through continuous changes. The size of the classifier recognizes objects of different sizes. At the same time, we set the identification target ID and divide the diabetic foot data into three categories: Blocked blood vessel id: 0, Suture id: 1, and Ulceration id: 2.

### 3.6. Module training

Before training, we will divide into training data sets and test data sets, with a quantity of 3:1. Using three different deep neural networks for feature search, the experiment using Fast R-CNN ResNet101 establishes a deep learning and Inception neural network structure model. Adjusting the parameters and the node layer makes the model more suitable for the diabetic foot wound for training. We set the training times around one hundred thousand times by observing the Tensorboard learning curve until it meets the minimum loss value and no longer changes. After successfully joining the data source, the additional panel has made the indicators visual output according to the required of the corresponding syntax query.

### 3.7. Determination of ulcer characteristics

The last stage of wound identification is carried out by classical image processing methods, including GrabCut and SURF algorithms, which are reviewed as follows. Once the bounding boxes containing the wounds are determined, the GrabCut and SUFT algorithms are applied to each sub-image inside the bounding box to determine each wound's boundaries and the transform matrix. Finally, the associated characteristics, such as the area and perimeter of each wound, are then computed.

#### 3.7.1. GrabCut algorithm

The GrabCut algorithm uses texture (color) information and boundary (contrast) information in the image, as long as a small amount of user interaction can get better segmentation results (34). The GrabCut algorithm is using iterative energy minimization, and thus, it is recognized as an iterative Graph Cut. The GrabCut algorithm uses the Gauss mixture model to model the target and background energy and perform the energy minimization iteratively to better image segmentation.

#### 3.7.2. SURF technique

Feature detection is the process of computing the image information's abstraction and making a local decision at every image point to see if there is an image feature of the given type existing at that point. Scale Invariant Feature Transform (SIFT) is a feature detector developed by Lowe (35). Although SIFT has proven to be very efficient in object recognition applications, it requires a large computational complexity which is a major drawback, especially for real-time applications. Speed up Robust Feature (SURF) technique, which is an approximation of SIFT, performs faster than SIFT without reducing the detected points' quality (36).

**Figure 5:**
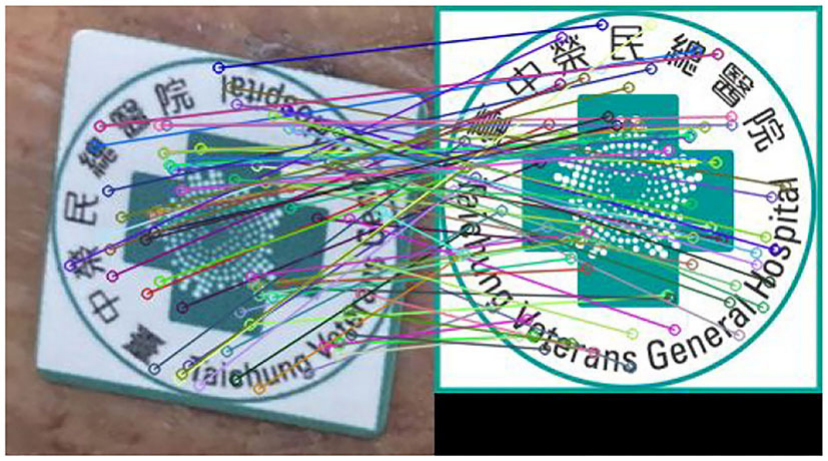
Feature points detected by using the SURF technique. The left subimage is the marker snapped from a typical wound image and the right image is the reference marker.

The SURF technique is used to identify the marker's feature points inside the wound image to its reference counterpart. Figure 5 shows the feature points between two types of marker images detected by the SUFT technique. The corresponding coordinates of these two images are used to calculate the transform matrix to denote the projective transformation from the left to the right images. In order to avoid the spacial discrepancies of the foot's concave wounds, the app will indicate the marker location such that the photograph is adequate for further analysis.

#### 3.7.3. Wound characteristics

**Figure 6:**
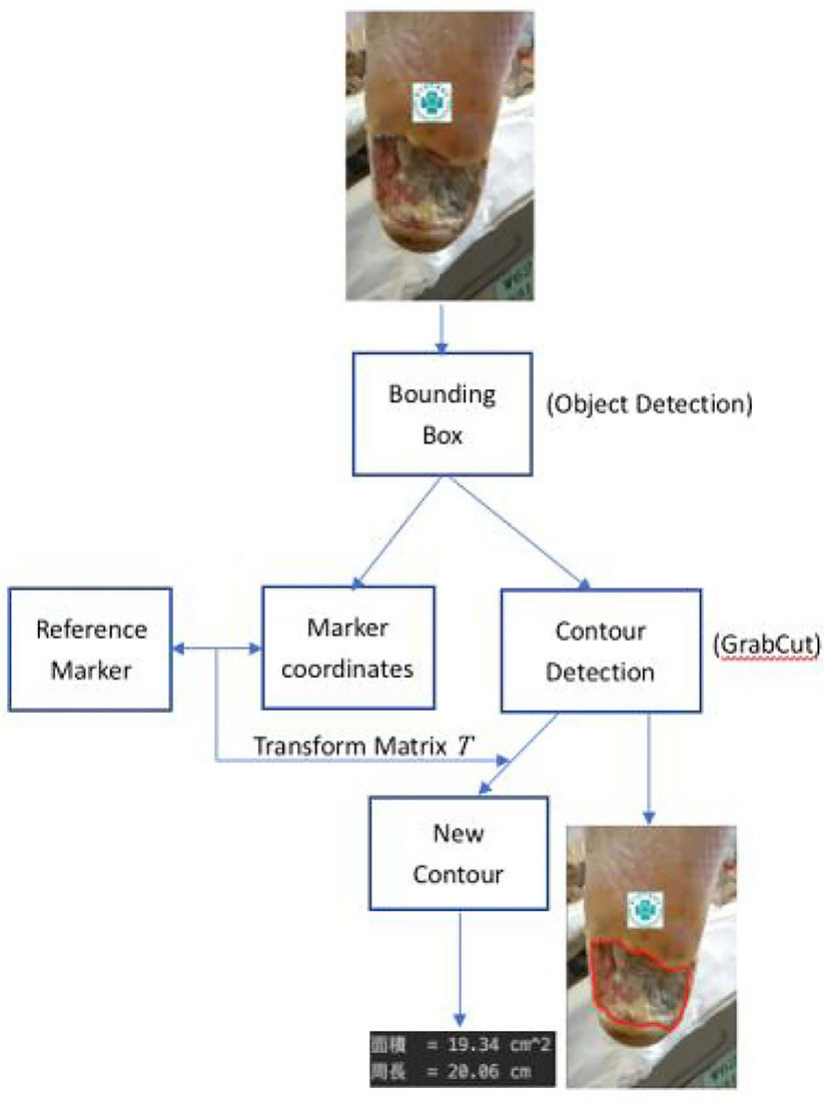
Wound characteristics computation process.

The schematic diagram to determine the characteristics of wounds is described in Figure 6.

Let *X*_*B*_*i*__, *i* = 1, 2, …, *m*, denote the portion of the image *X*_0_ which lies inside the *i*-th detected bounding box. Here *m* counts the number of ulcers inside *X*_0_. Also, let *X*_*M*_ denote the sub-image of *X*_0_ inside the bounding box containing the image of the reference marker. First, the histogram of these images is adjusted. Second, coordinates of corresponding points between the reference marker and the *X*_*M*_ are recorded as *z*_*R*_*M*__*i*__ = (*x*_*R*_*i*__, *y*_*R*_*i*__) and *z*_*M*_*i*__ = (*x*_*i*_, *y*_*i*_), *i* = 1, 2.…, *k*, by using the SURF algorithm as shown in Figure 5. Since the wound area in the sub=image *X*_*B*_*i*__ is recognized as a piece of foreground information, one then applies the Grabcut algorithm to segment *x*_*B*_*i*__ whose boundary is recorded as the contour, *X*_*C*_*B*__*i*__, of the corresponding wound with *i* = 1, 2, …, *m*.

Afterward, the projective matrix *T* which transforms from *z*_*M*_*i*__ to *z*_*R*_*M*__*i*__ is determined by solving the vector *A* to satisfy the equation.


(5) $$\begin{array}{r} \left[\begin{array}{cccccccc} x_{1} & y_{1} & 1 & 0 & 0 & 0 & -x_{1}x_{R_{1}} & -y_{1}x_{R_{1}}\\ 0 & 0 & 0 & x_{1} & y_{1} & 1 & -x_{1}y_{R_{1}} & -y_{1}y_{R_{1}}\\ x_{2} & y_{2} & 2 & 0 & 0 & 0 & -x_{2}x_{R_{2}} & -y_{2}x_{R_{2}}\\ 0 & 0 & 0 & x_{2} & y_{2} & 1 & -x_{2}y_{R_{2}} & -y_{2}y_{R_{2}}\\ \vdots & \vdots & \; & \; & \; & \; & \vdots \\ x_{k} & y_{k} & 1 & 0 & 0 & 0 & -x_{k}x_{R_{k}} & -y_{k}x_{R_{k}}\\ 0 & 0 & 0 & x_{k} & y_{k} & 1 & -x_{k}y_{R_{k}} & -y_{k}y_{R_{k}} \end{array}\right]A=\left[\begin{array}{c} x_{R_{1}}\\ y_{R_{1}}\\ x_{R_{2}}\\ y_{R_{2}}\\ \vdots \\ x_{R_{k}}\\ y_{R_{k}} \end{array}\right] \end{array}$$

and then the transform matrix is given by


(6) $$\begin{array}{r} T=\left[\begin{array}{ccc} A_{1} & A_{2} & A_{3}\\ A_{4} & A_{5} & A_{6}\\ A_{7} & A_{8} & 1 \end{array}\right] \end{array}$$

and the new contours *X*_*RC*_*B*__*i*__ = *TX*_*C*_*B*__*i*__ are computed in the reference coordinate, *i* = 1, 2, …, *m*. Finally we calculate the associated area and length of each new contours *X*_*RC*_*B*__*i*__, *i* = 1, 2…, *m*.

## 4. Experimental results

### 4.1. Image-assisted diagnosis of diabetic foot wounds

#### 4.1.1. Object category detection result

**Figure 7:**
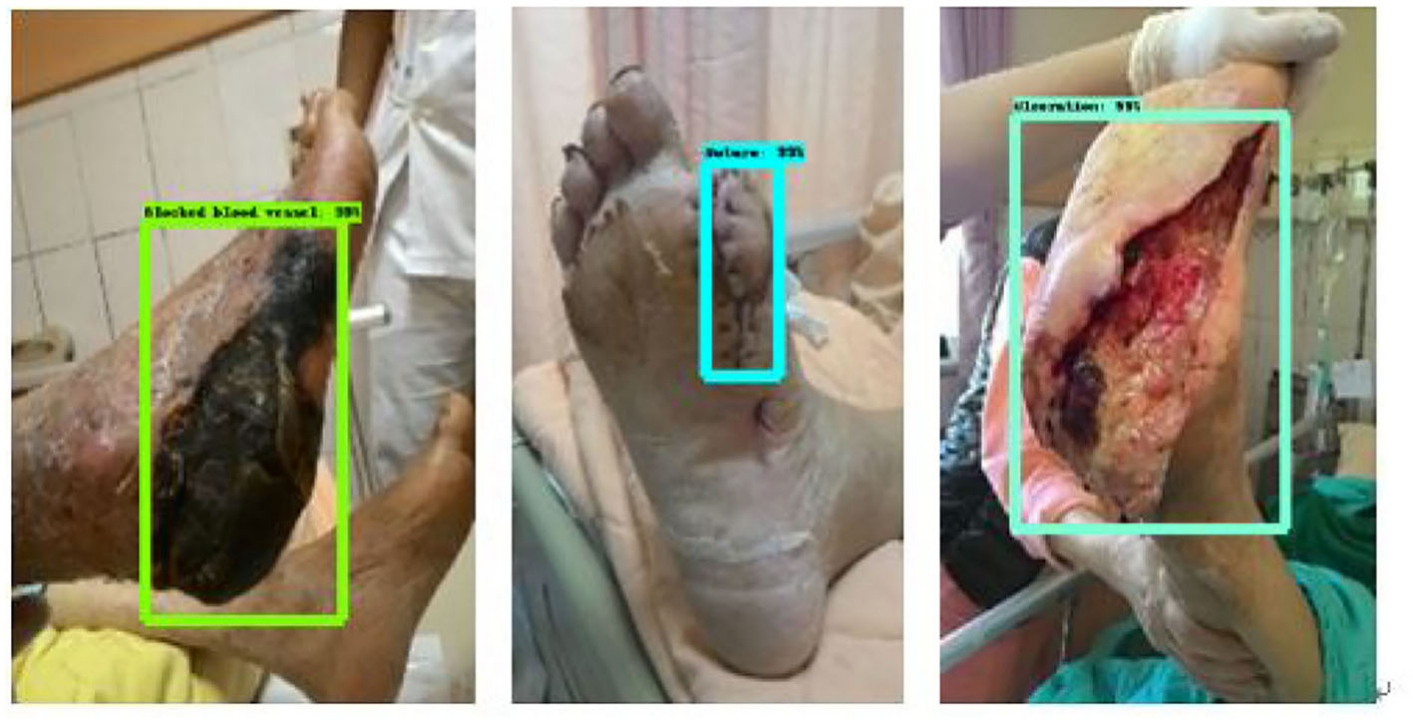
Identification results figure.

In this paper, three Fast RCNN modules were selected for migration, namely Inception v2-coco, Kitti-trained models (ResNet101), and iNaturalist species-trained models (ResNet101). Diabetic foot wound was trained. After 100,000 times of training, we observed the training curve through Tensorboard until the loss value reached the minimum and did not change. Figure 7 illustrates the three categories of module identification test results.

#### 4.1.2. Model comparison

After the training Inception v2-coco, Kitti-trained models (ResNet101), and iNaturalist species-trained models (ResNet 101), we compared the three modules. First, we compared the mAP precision and speed of the three modules. Of the three, ResNet101_Kitti showed the highest progress, up to 87. The maximum speed of ResNet101_fgvc was 395.

#### 4.1.3. Object detection training result

**Figure 8:**
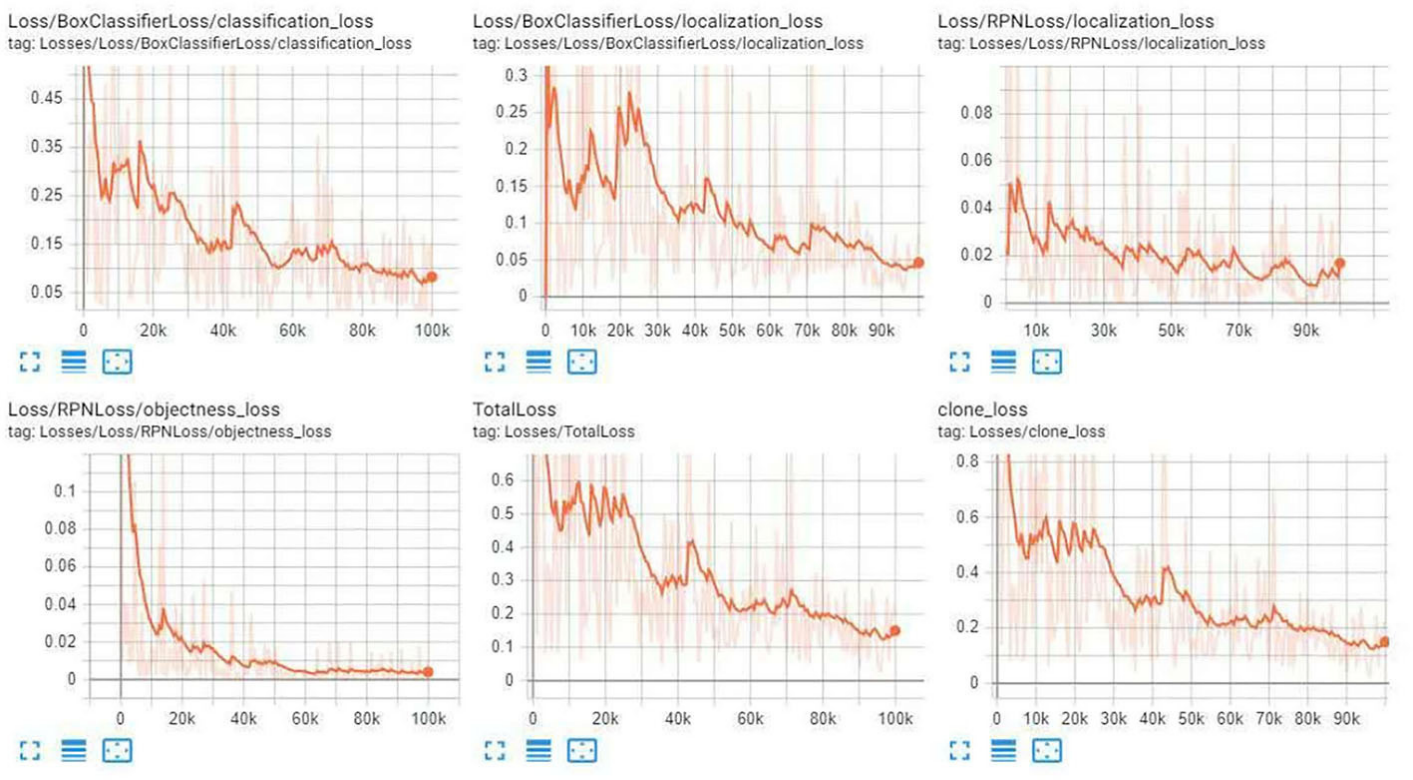
iNaturalist Species-trained models training loss graph.

**Figure 9:**
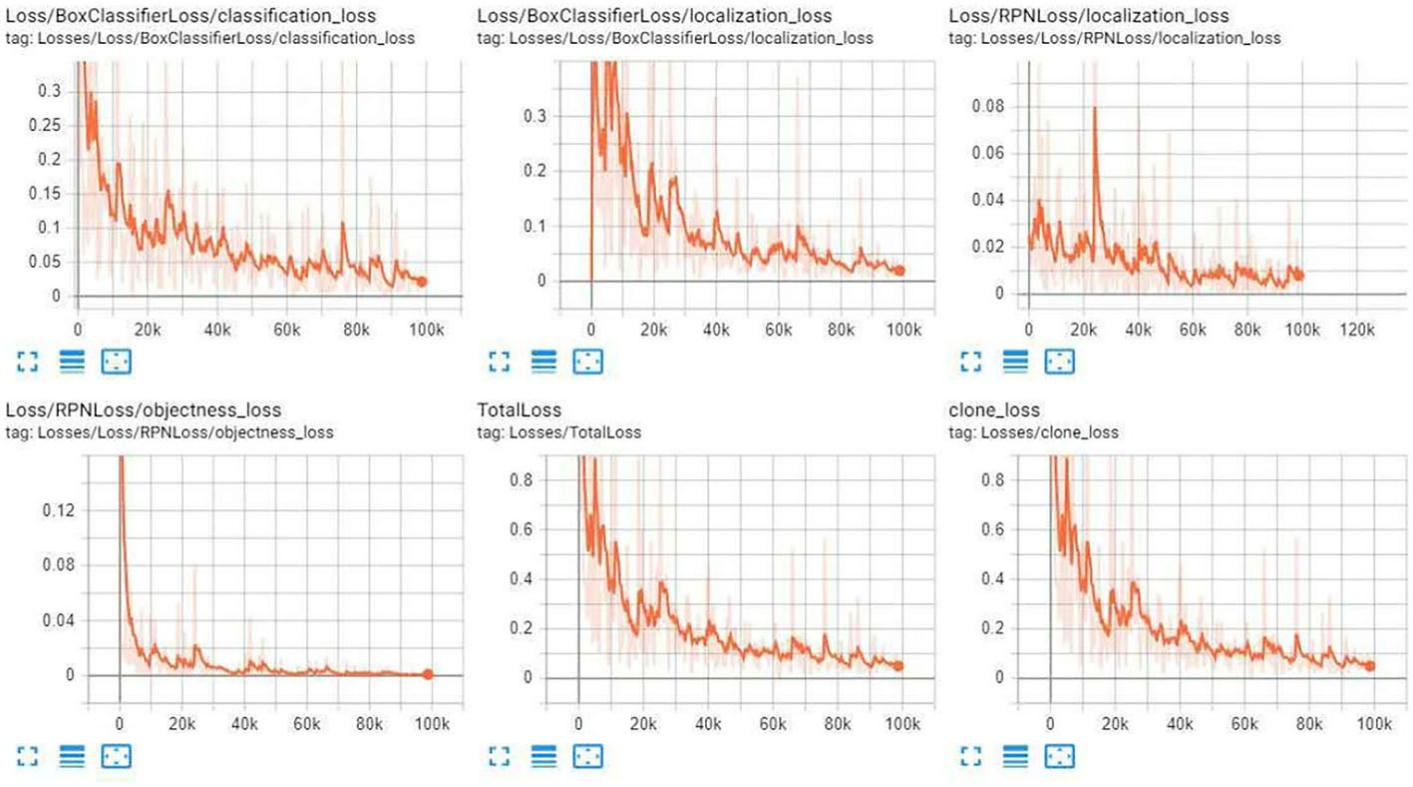
Kitti-Trained models (ResNet101) training Loss Graph.

**Figure 10:**
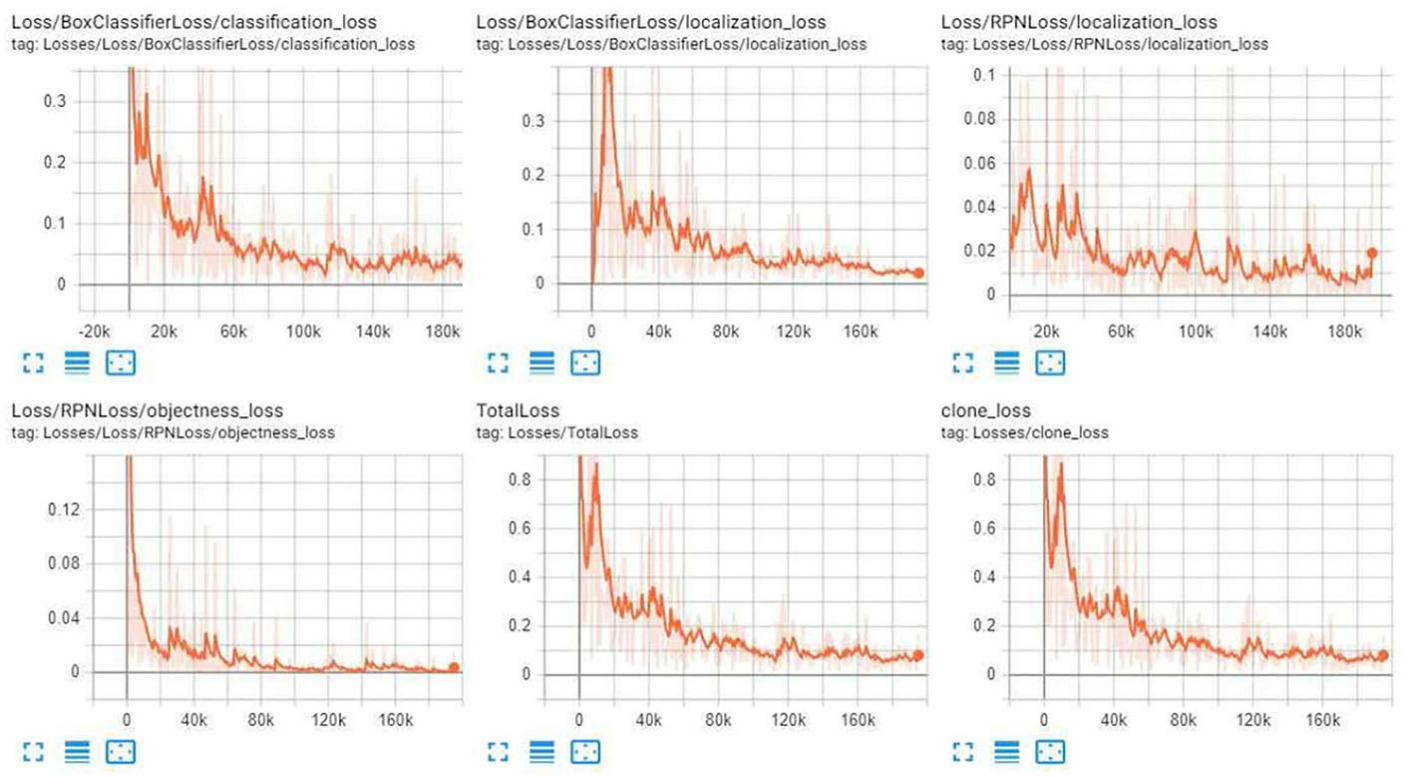
Inception V2-coco training loss graph.

Figures 8–10 illustrate the graph of the following models' training and loss:

1. iNaturalist Species-trained models

2. Kitti-Trained models (ResNet101)

3. Inception V2-coco

After the training, we compared the three modules. First, we compared the mAP precision and speed of the three modules as given in Tables 1–5. Of the three, ResNet101_Kitti showed the highest progress, up to 87. The maximum speed of ResNet101_fgvc was 395.

**Table 1:** iNaturalist species-trained models training loss value.

**Classification_Loss**	**Localization_Loss**	**TotalLoss**
0.05–0.10	<0.05	0.1–0.2

**Table 2:** Kitti-Trained models (ResNet101) training loss value.

**Classification_Loss**	**Localization_Loss**	**TotalLoss**
<0.05	<0.05	<0.1

**Table 3:** Inception V2- Coco training loss value.

**Classification_Loss**	**Localization_Loss**	**TotalLoss**
<0.05	<0.05	<0.1

**Table 4:** Comparison of Speed and mAP of different models.

**Model name**	**Speed (ms)**	**mAP**
faster_rcnn_inception_v2_coco	58	28
faster_rcnn_resnet101_kitti	79	87
faster_rcnn_resnet101_fgvc	395	58

**Table 5:** The accuracy rate of different models.

**Model**	**True**	**False**	**Total**	**Accuracy**
inception_v2_coco	87	13	100	87%
resnet101_kitti	88	12	100	88%
resnet101_fgvc	89	11	100	89%

Through the above charts and training curves, we found that from the perspective of the loss learning curve and mAP accuracy, Resnet101_kitti showed the highest learning accuracy. However, in our actual test, iNaturalist species-trained models Resnet101_fgvc showed the lowest background misdetection rate and wound identification accuracy of the photo. So we will use the iNaturalist species-trained models as the final module for wound identification.

#### 4.1.4. Calculate wound's characteristics

We cut the wound around the wound sub-image framed in the image through GrabCut and stroke it with a red line to mark the wound's contour. The average Hausdorff distance measures the sets denoting voxels of the ground truth and the proposed segmentation domain in our verification. If the segmentation is not acceptable to the physicians we adjust our parameters such that it covers the wounds. Eventually, 90% HD is used for our analysis which is acceptable by the physicians. The SURF technique is applied to identify the feature points between the marker sub-image and the referenced marker. The transform matrix is computed as stated in Section 3.7.3 and finds the reference coordinate's new contour. Once the rotational angle of the transform matrix is larger than 15°, the image is not analyzed further. Finally, evaluate the actual size of the wound in the reference coordinate.

**Figure 11:**
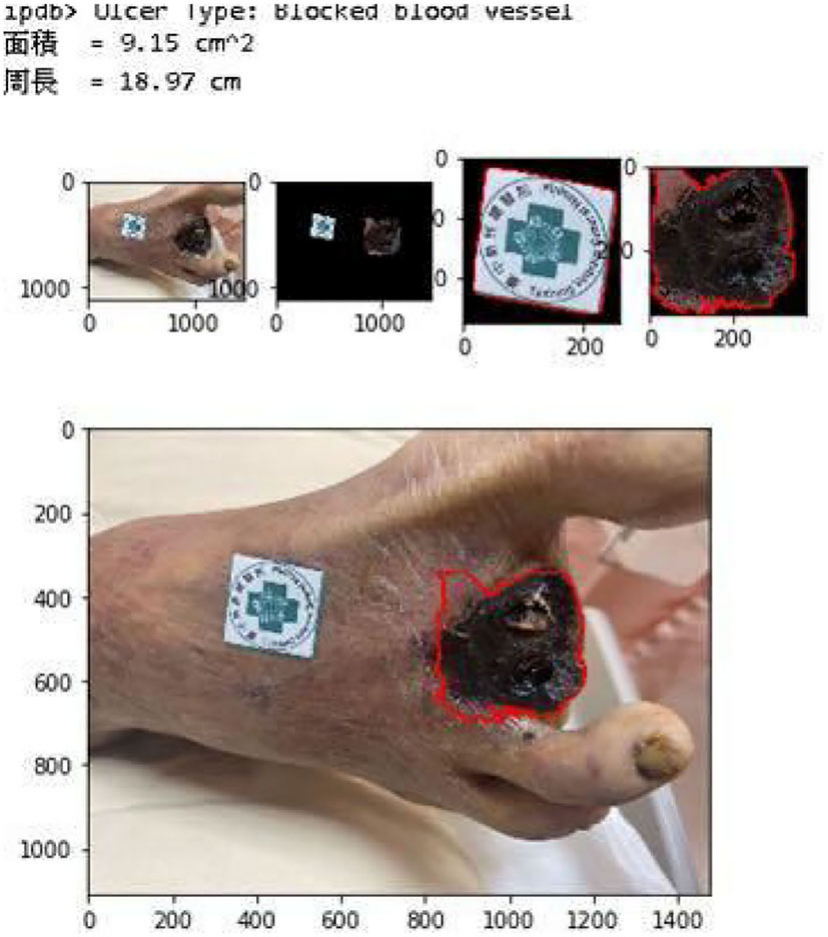
Wound characteristic computation. The top row shows the ulcer type information, and the area and perimeter of the wound. The middle row shows the segmentation of the wound and marker during our evaluation. The last row shows the contour marked with a red line.

**Figure 12:**
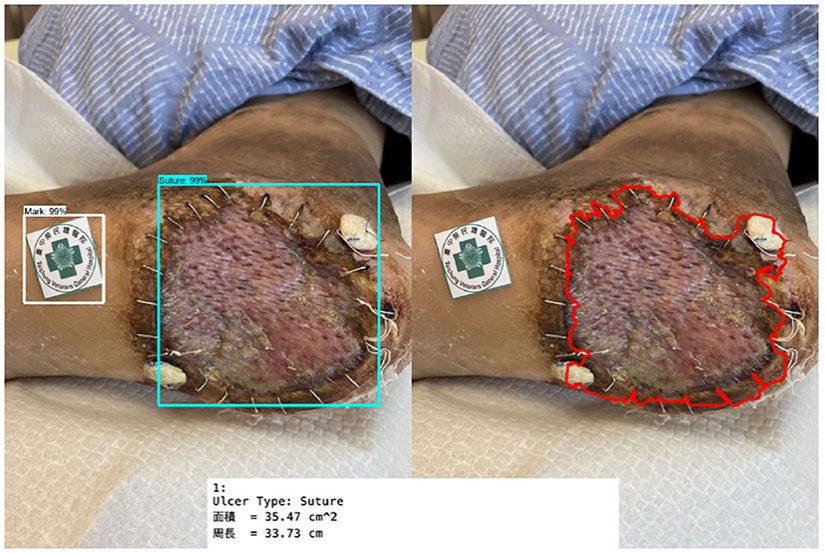
Wound contour identification for suture type of ulcers in a DFU foot.

**Figure 13:**
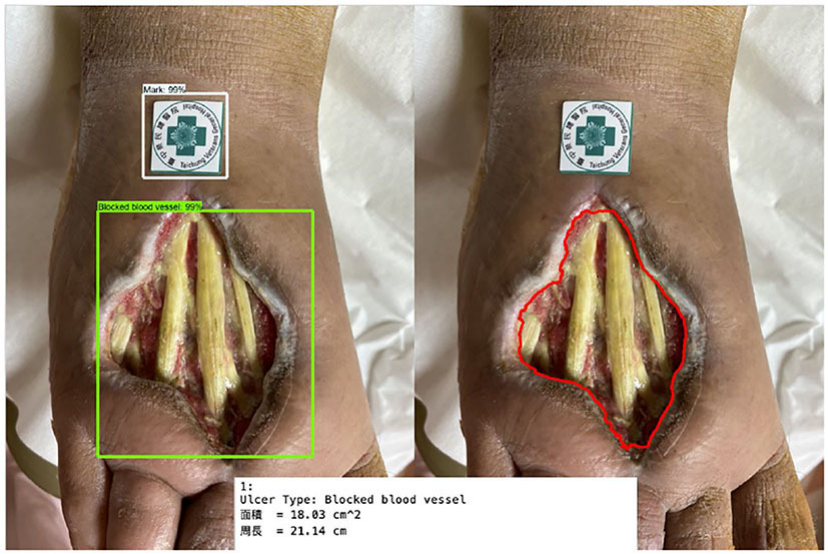
Wound contour identification for the ulcer type is not included in our learning process.

**Figure 14:**
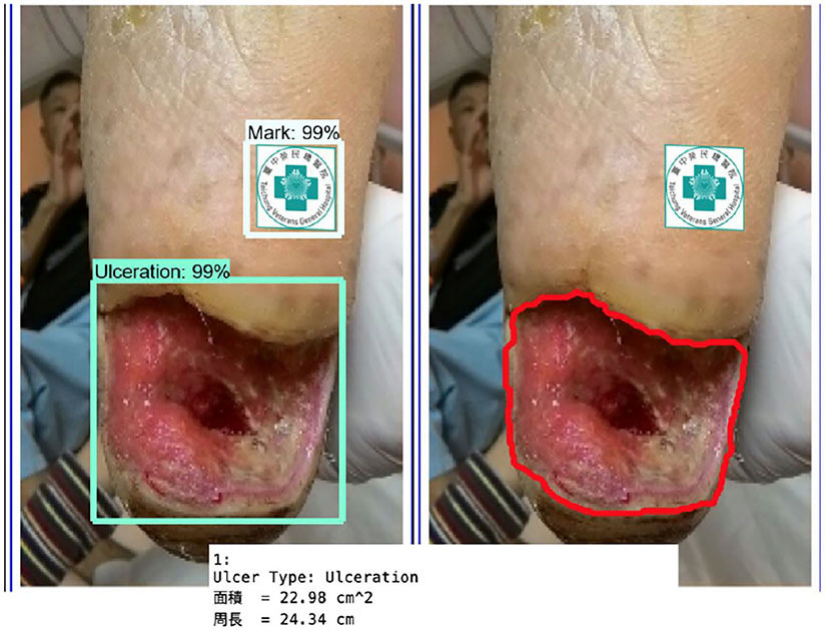
Wound contour identification for the ulceration type of a DFU foot after medical treatment.

Figure 11 shows the calculated area and perimeter of the wound. It is noted that the wound image also contains the image of the hospital's marker which served as a reference base for our calculation. Figures 12–14 show the wound contour identification for different types of ulcers in a DFU foot. It is noted that since the GrabCut algorithm contains the GMM information and the computation of iterative minimization which uses some random information and leads to not so accurate marked contour in the real application.

#### 4.1.5. Web page presentation

**Figure 15:**
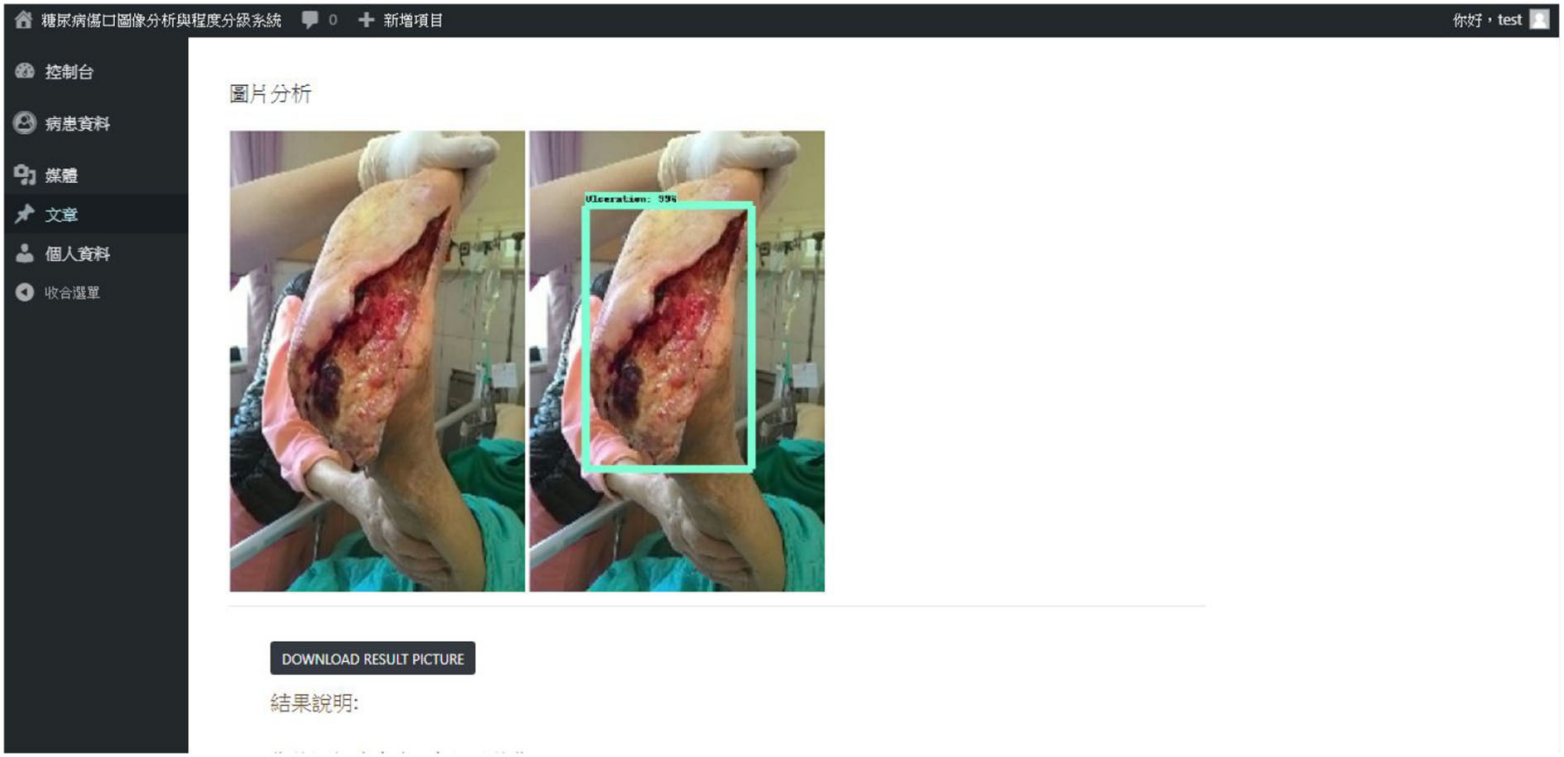
The webpage shows the identification analysis result.

This system uses WordPress for website writing, installs LAMP to connect to the network at the local location, connects the database, and completes the login page's interface. After successful login, the main page will be imported, and we can upload files or enter the image library. After the file is uploaded, we can enter the image library to view the record file. The uploaded image displays the results of the connector analysis on the page as shown in Figure 15.

The results of the analysis can be stored separately on the local side. We can delete uploaded images in the image library section.

**Figure 16:**
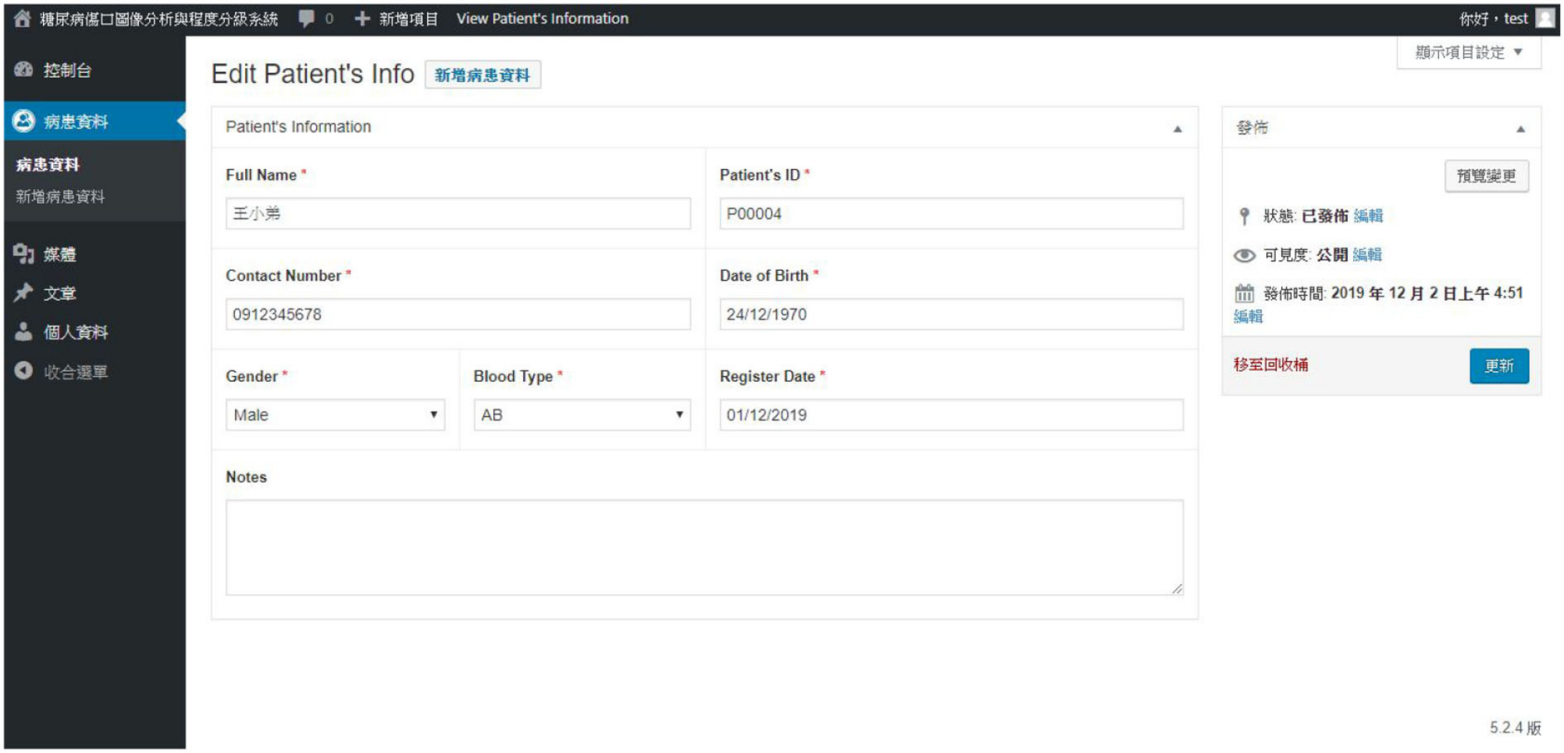
Another patient data query page.

By creating a patient profile, the user can further record the patient's name, gender, blood type, date of birth, and other information. Figure 16 describes patient data.

## 5. Conclusions and future works

This paper uses Object Detection to predict and classify different types of diabetic foot wounds and wound locations through a neural network. The system has currently extracted three categories (ulcers, sutures, blackening of the feet due to clogged blood vessels), analyzed, and classified the photos of diabetic foot ulcers. The wound characteristics such as area and perimeter are evaluated using GrabCut and SURF algorithms. To the best of our knowledge, this is the first successful experiment to automatically provide the wound's area and perimeter to fulfill the PEDIS evaluation index for the diabetic foot severity.

Users can import the images to the website. When there are multiple open wounds in the image, it will judge and mark the position and other wound information. In the training part, the iNaturalist Species-trained models (ResNet101) module is used to increase classifications by adjusting the parameters and changing the model component category. After evaluating each module of object detection's speed, we used Faster-RCNN-Inception-V2 and Faster-rcnn_ resnet101_kitti to make evaluations. Use iNaturalist Species-trained models (ResNet101) with fast speed and high mAP (Computer Vision Identification Accuracy) and low background false detection rate. Finally, in assessing accuracy, it was confirmed that the wound image detection data could be as high as 90%. On the other hand, due to the successful application of Mask-RCNN in the segmentation of wound images, the replacement of Faster-RCNN to Mask-RCNN will implement in the near future to provide more accurate segmentation.

In the future, relevant information will be added based on the existing wound classification, such as before and after wound comparison, and wound block range data, to provide more detailed records of physicians. Combine with electronic medical records and gradually increase the information of year and record, expand the richness of data, enhance data value, and make more accurate disease assessments for patients. Besides, the identification of wounds can extend from the current small range of a single wound to many different wound types. In the section of the website, we will gradually add more functions to enhance the utility, such as setting up the changing table of the patient, observing the function of comparing before and after the recovery process, expanding the richness of the data, and enhancing the value of the data. Also, the identification of wounds can determine multiple different types of wounds to be more stable, and it is expected that in the future. Users can provide a platform to evaluate the information of diseased foot wounds quickly through the improvement system.

## Data availability statement

The data presented in this article is not readily available because of ethical reasons. Requests to access the data should be directed to the corresponding authors.

## Ethics statement

The studies involving human participants were reviewed and approved by the Institutional Review Board of Taichung Veterans General Hospital (TCVGH), Taiwan (approval certificate number TCVGH-IRB Nos.: CE19340B and CE19340B-1). The patients/participants provided their written informed consent to participate in this study.

## Author contributions

H-NH, TZ, and C-TY contributed to the conception and design of the study. Y-JS and H-MC organized the database. H-MC and M-WT performed the statistical analysis. H-NH, C-TY, C-JC, and TZ wrote sections of the manuscript. All authors contributed to manuscript revision, read, and approved the submitted version.

## Funding

This work was funded in part by the Ministry of Science and Technology (MOST), Taiwan, under Grant Nos. 110-2221-E-029-020-MY3, 110-2621-M-029-003, 110-2622-E-029-003, 110-2115-M-029-002, 111-2622-E-029-003, and 111-2621-M-029-004. In addition, this work also funded by Taichung Veterans General Hospital (TCVGH), Taichung, Taiwan, under Grant Nos. TCVGH-T1087812, TCVGH-T1087813, and TCVGH-T1117806.

## Conflict of interest

The authors declare that the research was conducted in the absence of any commercial or financial relationships that could be construed as a potential conflict of interest.

## Publisher's note

All claims expressed in this article are solely those of the authors and do not necessarily represent those of their affiliated organizations, or those of the publisher, the editors and the reviewers. Any product that may be evaluated in this article, or claim that may be made by its manufacturer, is not guaranteed or endorsed by the publisher.
